# Conservation genetics of the threatened plant species *Physaria filiformis* (Missouri bladderpod) reveals strong genetic structure and a possible cryptic species

**DOI:** 10.1371/journal.pone.0247586

**Published:** 2021-03-11

**Authors:** Christine E. Edwards, Brooke C. Tessier, Joel F. Swift, Burgund Bassüner, Alexander G. Linan, Matthew A. Albrecht, George A. Yatskievych

**Affiliations:** 1 Center for Conservation and Sustainable Development, Missouri Botanical Garden, St. Louis, Missouri, United States of America; 2 Department of Cellular and Molecular Biology, Winona State University, Winona, Minnesota, United States of America; 3 Department of Biology, Saint Louis University, St. Louis, Missouri, United States of America; 4 Plant Resources Center, University of Texas at Austin, Austin, Texas, United States of America; University of Bordeaux, FRANCE

## Abstract

Understanding genetic diversity and structure in a rare species is critical for prioritizing both *in situ* and *ex situ* conservation efforts. One such rare species is *Physaria filiformis* (Brassicaceae), a threatened, winter annual plant species. The species has a naturally fragmented distribution, occupying three different soil types spread across four disjunct geographical locations in Missouri and Arkansas. The goals of this study were to understand: (1) whether factors associated with fragmentation and small population size (i.e., inbreeding, genetic drift or genetic bottlenecks) have reduced levels of genetic diversity, (2) how genetic variation is structured and which factors have influenced genetic structure, and (3) how much extant genetic variation of *P*. *filiformis* is currently publicly protected and the implications for the development of conservation strategies to protect its genetic diversity. Using 16 microsatellite markers, we genotyped individuals from 20 populations of *P*. *filiformis* from across its geographical range and one population of *Physaria gracilis* for comparison and analyzed genetic diversity and structure. Populations of *P*. *filiformis* showed comparable levels of genetic diversity to its congener, except a single population in northwest Arkansas showed evidence of a genetic bottleneck and two populations in the Ouachita Mountains of Arkansas showed lower genetic variation, consistent with genetic drift. Populations showed isolation by distance, indicating that migration is geographically limited, and analyses of genetic structure grouped individuals into seven geographically structured genetic clusters, with geographic location/spatial separation showing a strong influence on genetic structure. At least one population is protected for all genetic clusters except one in north-central Arkansas, which should therefore be prioritized for protection. Populations in the Ouachita Mountains were genetically divergent from the rest of *P*. *filiformis*; future morphological analyses are needed to identify whether it merits recognition as a new, extremely rare species.

## Introduction

As natural habitats continue to be lost and degraded as the result of anthropogenic stressors, many plant species are becoming increasingly threatened [1,2]. For the most rare and endangered plant species, in-depth research into their biology is often necessary to prevent their extinction and to ensure that they are adequately conserved. Population genetics is one avenue of research that can be particularly important for informing conservation efforts for rare species because it provides information about levels and structuring of genetic diversity, which can be used to devise in situ and ex situ conservation strategies. Ensuring that populations retain high genetic diversity is important because it is associated with increased individual-level fitness [3,4], greater potential for a species to adapt to environmental change [5], and increased ecosystem stability, resilience, and function [6–8].

Effective conservation requires knowledge of biodiversity, and population genetics is particularly useful for conservation because it can provide information about biodiversity at a variety of hierarchical levels. Estimates of genetic diversity within populations of a rare species can be compared to those of closely related species with similar life history characteristics to identify populations that have experienced losses in genetic diversity through processes associated with small population size and fragmentation, such as inbreeding, genetic drift, and genetic bottlenecks [9–11]; such information may therefore indicate populations in need of intensive conservation efforts to halt the loss of genetic diversity. Analyses to understand genetic structure and the partitioning of genetic diversity within and among populations can be used to help understand life history attributes (such as mating systems and breeding systems [12–20]), migration and habitat connectivity [21–25], and the demographic history of populations [26–28]. Using both neutral markers and those under selection, genetic data can be used to delineate conservation units [29,30], which can help facilitate the design of strategies to most effectively conserve the genetic diversity of a rare species in the face of limited resources [31,32]. Genetic data can also be used to help resolve species boundaries, such as determining whether a rare species is genetically unique and worthy of protection or whether it is instead a subpopulation of a more widespread species [33,34]. Genetic data may also unexpectedly reveal that some populations are genetically unique and may be cryptic species [35,36], in which case both the new species and the existing one may be in need of conservation. All of this information is important to ensure that diversity is being effectively conserved.

In this study, we focused on understanding genetic diversity in *Physaria filiformis* (Rollins) O’Kane & Al-Shehbaz (Missouri bladderpod; Brassicaceae), a threatened, herbaceous plant species with a winter-annual life history strategy [37,38]. *P*. *filiformis* is endemic to Missouri (MO) and Arkansas (AR), U.S.A. and occupies sparsely vegetated, glade habitats with shallow soils and exposed bedrock [37–40]. *P*. *filiformis* is fire maintained; when populations of *P*. *filiformis* are managed with controlled summer or early autumn burns, they often produce denser and larger flowering populations, although the response likely varies depending on the timing, frequency, and intensity of fire [41]. Fire-suppressed populations exhibit large decreases in size because *P*. *filiformis* are small in stature and are easily over-topped and outcompeted by other grass and forb species [38,42]. Because *P*. *filiformis* has an annual lifecycle, populations demonstrate large year-to-year fluctuations in population size, associated not only with fire but also likely with a range of ecological and climatic conditions [43]. In addition to threats related to fire suppression, the species is threatened by habitat loss as the result of overgrazing, residential and commercial development, spread of exotic species, and unsuitable land management practices [40,43].

**Fig 1:**
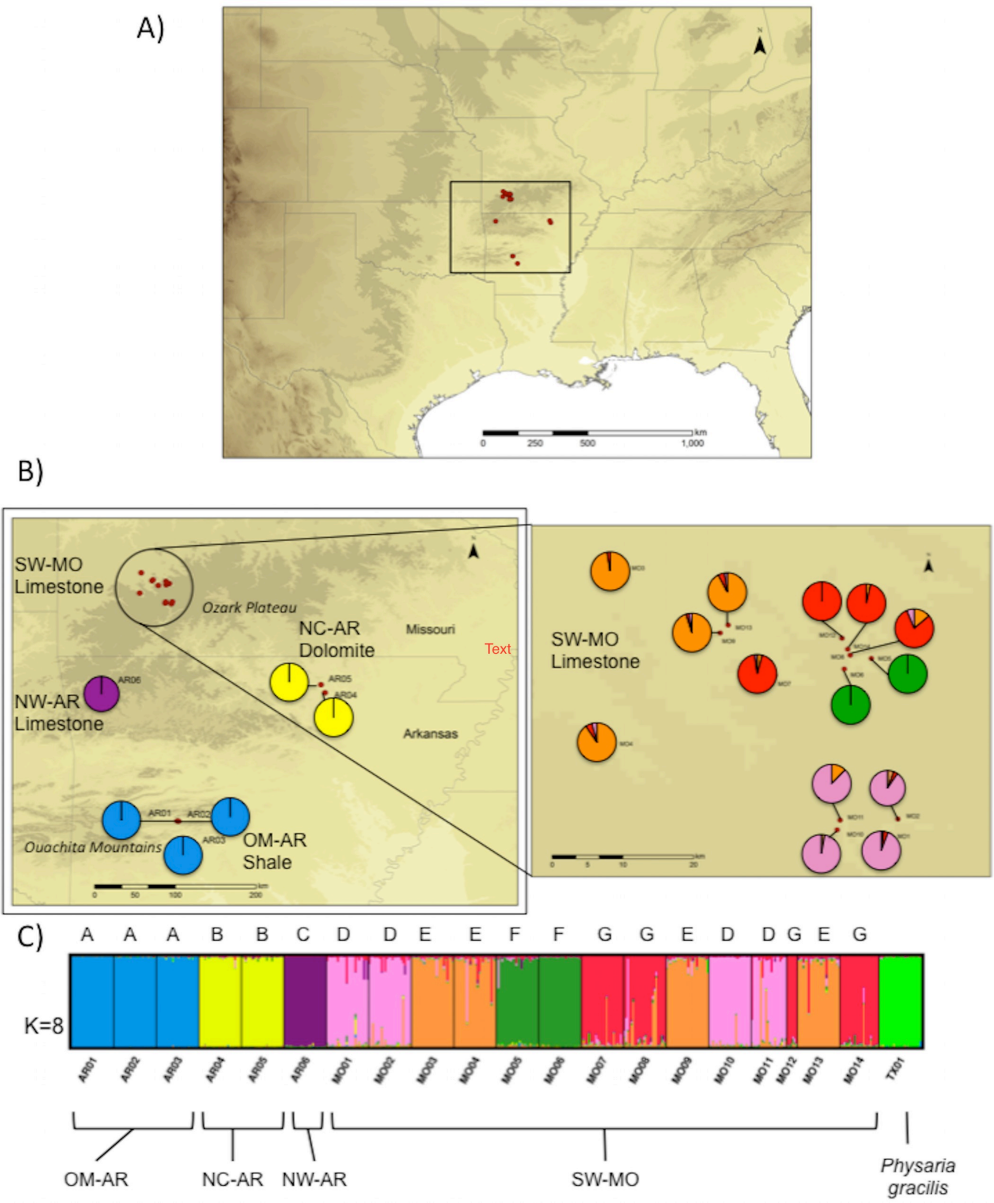
Map of the distribution and collection localities of *P*. *filiformis* and results of STRUCTURE analysis. A) The larger map shows the distribution of *Physaria filiformis* in North America, with the square indicating the area shown in Fig 1B (left). B) The map (left) shows sampling locations of *P*. *filiformis* in MO and AR with the main geographic groups and their soils labeled. The inset (right) shows the sampling locations within MO. The three more northern population groups occupy the Ozark Plateau physiographic region, whereas the southernmost population group occupies the Ouachita Mountains. Brown colors indicate elevation, with darker brown indicating areas of lower elevation (layers available at http://www.diva-gis.org/gdata). Dots or pie charts indicate the collection localities of the 20 populations of *Physaria filiformis* sampled in the study. Pie charts indicate how populations were assigned to genetic clusters by STRUCTURE. C) Results of STRUCTURE analyses for *P*. *filiformis* and *P*. *gracilis* at *K* = 8. Populations are separated by black lines. Each vertical line within a population represents an individual, the genetic clusters are represented by a unique color, and the proportion of membership of each individual in genetic clusters is indicated by the colors of each line. The four main geographic regions are indicated at the bottom of Fig 1C. OM-AR, Ouachita Mountains, Arkansas; NC-AR, north-central Arkansas; NW-AR, northwestern Arkansas; SW-MO, southwestern Missouri.

*P*. *filiformis* was listed as Endangered under the U.S. Endangered Species Act (ESA) in 1987, at which time it was known from only nine sites in southwestern MO [40,43]. Subsequently, dozens of additional populations were discovered, and *P*. *filiformis* was down-listed to threatened under the ESA in 2003 [44]. Currently, the species is known from 67 sites occupy in four distinct geographic areas in MO and AR (Fig 1A). Three of the four groups are located in the Ozark Plateau physiographic region, including: 1) a group of populations in southwest MO (SW-MO), 2) a single population in northwest AR (NW-AR), and 3) a small group of populations in north-central AR (NC-AR) [37,43, Fig 1 and S1 Table]. The final group, which consists of three populations, occupies the Ouachita Mountains physiographic region (OM-AR) [37,43, Fig 1 and S1 Table]. The soil substrate of the four regions varies; the three more Ozark Plateau populations occupy calcareous substrates, with the populations in SW-MO and NW-AR occurring on limestone and the NC-AR populations occurring on dolomite, whereas the OM-AR populations occupy sites with shale bedrock [37,43,45, Fig 1 and S1 Table]. Whether populations are adapted to these soil substrates is unknown, but it is possible that substrate could have acted as a selective force shaping the evolutionary history of the species.

Given the increase in the known number of populations, *P*. *filiformis* is now considered a potential candidate for de-listing. The criteria for de-listing outlined in the U.S. Fish and Wildlife Service’s (USFWS) recovery plan for Missouri bladderpod are the protection of 30 scattered, self-sustaining populations, 15 of which must be in public ownership [40]. However, these recovery criteria were developed when the species was known from only one of the four main geographic groups; one question that remains is whether the species exhibits genetic structure because it occupies two physiographic regions, has a naturally fragmented range, or because of the different soil types it inhabits [43], which have been proposed as factors that may shape genetic structure in the species [43]. Also unknown is whether the populations that are publicly protected encompass a broad range of the genetic variation present in the species. Although several previous genetic analyses were performed on *P*. *filiformis* [46–49], they each focused on a very small number of populations in Missouri. Results of these studies generally indicated that populations of *P*. *filiformis* have relatively high genetic diversity, consistent with its obligately outcrossing (self-incompatible) mating system [13,47]. However, no genetic analysis has yet been conducted that encompasses the known geographic range of *P*. *filiformis* it is unknown whether current conservation efforts are adequately protecting genetic diversity in the species.

The main goal of the study was to understand genetic diversity and structure in *Physaria filiformis* to inform conservation efforts. We developed 16 microsatellite markers for *P*. *filiformis* and used them to genotype populations sampled throughout the species’ range. We analyzed levels of genetic diversity in populations of *P*. *filiformis*, compared them to each other and to those of a close relative, *Physaria gracilis* (Hook.) O’Kane & Al-Shehbaz, to detect whether populations may have experienced losses in genetic diversity, and investigated genetic structure. The specific goals were to: (1) measure genetic diversity within populations and identify whether any factors associated with small population size, such as inbreeding, genetic drift or genetic bottlenecks, may have reduced levels of genetic diversity, (2) understand genetic structure and the factors affecting the structuring of genetic variation in *P*. *filiformis*, and (3) understand how much genetic variation of *P*. *filiformis* is currently publicly protected and develop *in situ* and *ex situ* conservation strategies to protect the full range of genetic diversity.

## Materials and methods

### Study species, sample collection, and DNA extraction

*P*. *filiformis* (Rollins) O’Kane & Al-Shehbaz is a winter annual plant in the Brassicaceae family that forms rosettes in the late fall or early winter, develops potentially numerous stems that bear flowers in the spring, and then completes its life cycle by June [37,40]. Stems are slender, branched, 4–8 inches tall, and densely covered with stellate hairs [39]. The species has bright yellow flowers with four petals that are clustered at the tops of the stems in indeterminate inflorescences [39]. Previous pollination studies showed that the species is insect-pollinated and likely has a genetic system of self-incompatibility (i.e., sporophytic self-incompatibility; [42]). The fruits of *P*. *filiformis* are round capsules ~1/8 inch in diameter (hence the common name bladderpod) that contain four small seeds, which are gravity or water-dispersed [37,39,40].

In 2015 and 2016, we sampled individuals from 20 populations of *P*. *filiformis* from throughout its known geographic range. All necessary private landowner permission and collection permits (from Missouri Department of Conservation, Missouri Department of Natural Resources, and federally owned sites under USFWS Permit # TE23537-1) were obtained prior to collection. Whenever possible, we sampled 24 individuals per site, although fewer individuals were sampled in three sites with smaller population sizes (S1 Table). When sampling within populations, we flagged all individuals occurring in each site and collected leaf tissue from individuals distributed evenly across the population. We also collected a single voucher specimen per population; vouchers were deposited in the MO and TEX herbaria (see S1 Table for voucher information). In SW-MO, where the majority of the known populations of *P*. *filiformis* occur, we sampled in each of 14 sites (S1 Table; Fig 1). Although we also visited an additional 11 sites in SW-MO where *P*. *filiformis* was observed previously, we were unable to locate it at these sites. In Arkansas, we sampled a total of six populations spread across three geographically separated areas, including one population in NW-AR, two populations in NC-AR, and three populations in OM-AR. In addition, we sampled a population of *Physaria gracilis* (a more widespread relative of *P*. *filiformis*) in Texas to understand how levels of genetic diversity in *P*. *filiformis* compared to a closely related congener. Although the phylogenetic relationship between *P*. *gracilis* and *P*. *filiformis* is unknown (no published phylogeny has included both species), we included *P*. *gracilis* because it is a morphologically well-differentiated congener and has the most geographically proximal distribution to *P*. *filiformis*. The final data set comprised a total of 480 individuals, with individuals sampled from 20 populations of *P*. *filiformis* and one population of *P*. *gracilis*. Leaf tissue was placed into silica gel for subsequent DNA extraction. Silica-dried leaf tissue was brought back to the lab, where DNA was extracted from each of the 480 individuals using a CTAB protocol [50].

### Microsatellite marker development and genotyping

We used shotgun sequencing of genomic DNA to identify microsatellites following the protocol described in Swift et al. [51]. Briefly, genomic DNA of two individuals of *P*. *filiformis* (from the AR01 and MO06 populations) was prepared for DNA sequencing using Nextera DNA sample prep kits and Nextera index kits (Illumina) and sequenced using 2×150 bp reads an Illumina MiSeq. The MiSeq run of the two samples resulted in 1,734,021 and 1,096,693 paired-end reads, which were deposited in GenBank (SRA bioproject number PRJNA675628). Reads were trimmed and assembled *de novo* into 601,797 contigs using the Medium sensitivity/fast setting using Geneious version 7.1.9 (Biomatters Inc.). Microsatellites were identified and polymerase chain reaction (PCR) primers were designed using MSATCOMMANDER [52] and PRIMER3 [52,53]. We added an M13 tag (5’-CACGACGTTGTAAAACGAC-3’) to the 5’ end of each forward primer to employ a universal dye-labeling approach [54]. MSATCOMMANDER identified 591 unique microsatellites in contig consensus sequences, from which PRIMER3 identified 148 unique microsatellite primers.

We initially tested 144 primers for amplification using genomic DNA from the same two samples of *P*. *filiformis* listed above using protocols described in Swift et al. [51]. PCR amplifications were performed in 10 μL reactions containing 0.5 U of GoTaq Flexi DNA polymerase (Promega), 1× Promega Colorless GoTaq Flexi Buffer, 1.5 mM MgCl_2_, 4.5 pmol each of the reverse primer and one of four fluorescently labeled M13 primers (6-FAM, VIC, NED, or PET; Applied Biosystems), 0.18 pmol of the M13-tagged forward primer, and 0.5 mM of each dNTP. PCR temperature cycling conditions were as follows: (I) 3 min at 94°C, (II) denaturation for 30 s at 94°C, (III) annealing for 30 s at 52°C, (IV) extension for 45 s at 72°C, (V) 35 repetitions of steps 2–4, and (VI) a final elongation at 72°C for 20 min. In this initial screening, 24 loci that amplified in both individuals of *P*. *filiformis* were selected for further testing in 8 individuals sampled from across the range of *P*. *filiformis* (1–2 individuals from each of six populations, including the AR01, AR03, AR04, MO05, MO06, and MO-07 populations). We selected 16 polymorphic loci with consistent amplification and easily scoreable peaks (S2 Table).

To assess patterns of genetic diversity and structure, we genotyped each of 480 sampled individuals at the 16 polymorphic microsatellite loci (S2 Table; S1 File). In PCR, four fluorescently labeled M13 primers, each labeled with a differently colored dye, were used to label separate loci, which were then pooled for analysis. All genotyping was carried out using capillary electrophoresis on an Applied Biosystems 3730xl at the DNA analysis facility at Science Hill at Yale University. Fragment analysis and scoring were conducted using automated fragment scoring panels developed for each locus in GeneMarker version 2.6.2 (Soft Genetics LLC), and then the data were checked manually.

### Data analysis

We tested for linkage disequilibrium (LD) between pairs of loci in each population and for deviations from Hardy-Weinberg equilibrium (HWE) at each locus/population combination using Fisher’s exact tests in Genepop [55]. Microsat Analyser [56] was used to calculate diversity indices and summary statistics, including average number of alleles per locus, observed and expected heterozygosity, and the inbreeding coefficient (*F*). Private alleles were calculated using GenAlEx [57]. FSTAT version 2.9.3.2 [58] was used to calculate allelic richness, using rarefaction to account for differences in sample size [59]. Because null alleles can cause large heterozygote deficiencies and inbreeding coefficients, we used INEST version 2.0 (Chybicki & Burczyk, 2009) to test for the presence of null alleles. INEST employs a population-inbreeding model to measure the frequency of null alleles at each locus while simultaneously calculating the inbreeding coefficient (*F*) within each population. We employed the Bayesian MCMC approach with 200,000 cycles, keeping every 200^th^ update, and a burn-in period of 20,000 cycles to calculate the percent of null alleles and a revised *F* (*F*^*B*^) in each population. The TX01 population was missing genotypic information at two loci (PF84 and PF121), such that only 14 loci were analyzed for this population.

To test whether any *P*. *filiformis* populations have undergone recent genetic bottlenecks, we used the program *BOTTLENECK* version 1.2.02, with the default parameters [60]. Because the detection of heterozygote excess may be sensitive to the mutation model employed [61,62], we tested for bottlenecks using several mutation models, including the infinite alleles model (IAM), the stepwise mutation model (SMM), and a mixed-model option (two-phase mutation model, or TPM) with 70%, 80%, and 90% of the loci assumed to be following the SMM. The one-tailed Wilcoxon signed rank test for heterozygote excess was used to assess the significance of these tests, and we considered populations to have undergone a recent bottleneck if significant results were obtained for tests using two or more mutation models [61].

To investigate patterns of genetic structure, we used Microsat Analyser to calculate pairwise *F*_*ST*_ for all possible pairs of populations, with 10,000 permutations to assess significance. Because *F*_*ST*_ values of highly variable markers, such as microsatellites, are constrained by population-level homozygosity levels and thus do not reach their maximum at 1 (see Hedrick 2005 for a more detailed explanation), we also calculated a standardized measure of pairwise population differentiation, *G’*_*ST*_ [63] using the program Genodive [64]. We used sequential Bonferroni corrections for all tests involving multiple comparisons [65]. Additionally, we used Mantel tests to analyze whether populations of *P*. *filiformis* exhibited isolation by distance [66]. We calculated a pairwise genetic distance matrix in Genodive using Nei’s [67] genetic distances, generated a pairwise geographic distance matrix using the geographic distance calculator [68], and analyzed the correlation between the matrices using a standard Mantel test with 10,000 permutations in Genodive. For a graphical representation of population structure, we generated a neighbor joining tree of Nei’s standard genetic distance in PAUP 4.0a159 [69].

To investigate patterns of genetic structure in *P*. *filiformis* without *a priori* grouping of individuals into populations, we analyzed the data using STRUCTURE version 2.3.4 [70]. We varied the number of groups in the data set (*K*) and assigned admixture proportions of each individual to these groupings. We used an admixture model, assumed correlated allele frequencies, used a flat prior of *K*, and did not incorporate population information into the analyses. We ran analyses following the recommendations of Gilbert et al. [71]; after preliminary analyses to determine the adequate burn-in and number of iterations, we ran 10 separate runs at each *K* from 1 to 15, with a burn-in of 500,000 generations and a run length of 1,000,000 generations. We examined the groupings across all runs at each *K* to ensure that the results were consistent [71] using clumpak [72,73]. To determine the appropriate value of *K*, we plotted the average natural logarithmic probability (-ln prob) of the data across multiple replicates of each *K* using structure harvester [74], and selected the value of *K* at which the (-ln prob) began to plateau. To determine priorities for *ex situ* conservation, we identified the populations within each STRUCTURE cluster with the greatest number of private alleles.

Analysis of molecular variance (AMOVA) was conducted using Arlequin version 3.5.2.2 [75] to understand the hierarchical partitioning of genetic variation and to compare how much variation is explained using different population grouping strategies. We grouped populations according to 1) physiographic region (i.e., Ouachita Mountains or Ozark Plateau), 2) soil substrate (i.e., limestone, dolomite, or shale as listed in Table 1), 3) geographic area (i.e., the four main population localities shown in Fig 1 and indicated in Table 1), or 4) based on the genetic clusters identified using STRUCTURE (see Results below). We considered the optimal grouping strategy to be the one that explained the greatest amount of among-group variation.

**Table 1:** Population information, genetic diversity statistics and conservation priorities for *Physaria filiformis*.

Population	Geographic area*	Soil Type	n	*A*_*R*_	*A*	*A*_*P*_	*H*_*O*_	*H*_*E*_	*F*	% null	*F*^*B*^	Genetic cluster	Seed banking priority	Publicly protected *in situ*?	Priority for *in situ* conservation?
*P*. *filiformis*															
AR01	OM-AR	Shale	24	1.87	2.50	3	0.32	0.29	-0.11	0.02	0.02	A	1	yes	
AR02	OM-AR	Shale	24	2.04	2.81	1	0.31	0.32	0.03	0.05	0.04	A	2	yes	
AR03	OM-AR	Shale	24	2.43	3.50	5	0.39	0.42	0.07	0.02	0.05	A	1	no***	
AR04	NC-AR	Dolomite	24	2.47	3.38	1	0.32	0.43	0.25	0.16	0.09	B	2	no	yes
AR05	NC-AR	Dolomite	24	2.55	3.75	2	0.37	0.44	0.17	0.05	0.06	B	1	no	yes
AR06	NW-AR	Limestone	24	2.52	3.31	4	0.44	0.47	0.07	0.05	0.03	C	1	yes	
MO01	SW-MO	Limestone	24	2.80	4.25	1	0.49	0.51	0.05	0.09	0.03	D	1**	no	
MO02	SW-MO	Limestone	24	2.90	4.38	0	0.46	0.52	0.11	0.05	0.04	D	3	no	
MO03	SW-MO	Limestone	24	2.81	4.75	2	0.42	0.47	0.11	0.06	0.04	E	1	yes	
MO04	SW-MO	Limestone	24	2.53	3.81	0	0.40	0.42	0.05	0.06	0.04	E	3	no	
MO05	SW-MO	Limestone	24	2.78	4.31	1	0.42	0.48	0.14	0.12	0.07	F	1**	no	
MO06	SW-MO	Limestone	24	2.43	3.50	1	0.36	0.42	0.16	0.07	0.04	F	1**	yes	
MO07	SW-MO	Limestone	24	2.54	3.88	2	0.41	0.44	0.07	0.11	0.05	G	1	yes	
MO08	SW-MO	Limestone	24	2.96	5.06	0	0.47	0.50	0.06	0.04	0.06	G	3	yes	
MO09	SW-MO	Limestone	24	3.23	5.81	1	0.51	0.55	0.06	0.01	0.04	E	2	no	
MO10	SW-MO	Limestone	24	2.53	3.63	1	0.40	0.46	0.13	0.04	0.06	D	1**	yes	
MO11	SW-MO	Limestone	20	2.76	4.56	1	0.41	0.50	0.17	0.02	0.09	D	1**	yes	
MO12	SW-MO	Limestone	6	2.48	2.75	0	0.34	0.42	0.21	0.13	0.08	G	3	no	
MO13	SW-MO	Limestone	24	3.13	5.19	2	0.48	0.53	0.10	0.06	0.04	E	1	yes	
MO14	SW-MO	Limestone	22	2.83	4.31	0	0.44	0.50	0.11	0.05	0.05	G	3	yes	
Average				2.63	3.97		0.41	0.45	0.10	0.06	0.05				
*P*. *gracilis*		Mudstone													
TX01	TX		24	1.54	4.33	6	0.38	0.51	0.27	0.17	0.02				

*OM-AR, Ouachita Mountains, Arkansas; NC-AR, north-central Arkansas; NW-AR, northwestern Arkansas; SW-MO, southwestern Missouri.

*Indicates that several populations have equal numbers of private alleles and at least one of the populations should be sampled per genetic cluster.

**Indicates populations that are not publicly protected but are protected for conservation purposes by the landowner.

Characteristics of populations include geographic area and soil type. Genetic diversity statistics as measured across 16 microsatellite loci include sample size (n); average allelic richness (*A*_*R*_); average number of alleles per locus (*A*); total number of private alleles (*A*_*P*_), average observed heterozygosity (*H*_*O*_); average expected heterozygosity (*H*_*E*_), inbreeding coefficient (*F*), % null percent null alleles; and the inbreeding coefficient taking into account null alleles (*F*^*B*^). Genetic cluster represents the groups recovered at *K* = 8 in STRUCTURE (see Fig 1B). Seed banking priorities were ranked according to the greatest private alleles in each STRUCTURE cluster, with 1 = high priority and 3 = low priority. *In situ* conservation priorities were evaluated based on whether at least one population was protected in each STRUCTURE cluster.

## Results

### Levels of genetic diversity in populations of *P*. *filiformis*

The mean number of alleles across all loci (*A*) in each population ranged from 2.50–5.81 (overall mean = 3.97; Table 1) and allelic richness (*A*_*R*_) ranged from 1.87–3.23 (overall mean = 2.63; Table 1). Both *A*_*R*_ and *A* were markedly lower in two populations in the Ouachita Mountains, AR01 and AR02, than all other populations of *P*. *filiformis* (Table 1). The number of private alleles in each population ranged from 0–5, with the greatest numbers found in the AR01, AR03, and AR06 populations. The *H*_*o*_ (observed heterozygosity) averaged across all loci ranged from 0.31–0.51 (overall mean = 0.408; Table 1) and *H*_*E*_ (expected heterozygosity) ranged from 0.29–0.55 (overall mean = 0.45; Table 1) in *P*. *filiformis*. The inbreeding coefficient in *P*. *filiformis* ranged from -0.11–0.25 (overall mean = 0.10). The percentage of null alleles in each population averaged across all loci ranged from 0.01–0.16 (overall mean = 0.06; Table 1). All loci consistently had a small frequency of null alleles across the populations of *P*. *filiformis*, and when these were taken into account, the inbreeding coefficients (*F*^*B*^*)* were very close to zero, ranging from 0.02–0.09 (overall mean = 0.05; Table 1). Correspondingly, populations did not show significant deviations from Hardy-Weinberg equilibrium. In comparison to the single population of the congener *P*. *gracilis*, values of *A*, *H*_*o*_, and *H*_*E*_ in *P*. *filiformis* were generally comparable, while values of *A*_*R*_ were greater and *F* were smaller. However, the lower *A*_*R*_ and greater *F* values in *P*. *gracilis* are likely because it had the greatest percentage of null alleles (17%; Table 1) and showed overall lower amplification success. Indeed, when null alleles were taken into account, *F* values in *P*. *gracilis* decreased from 0.27 to 0.02 (Table 1).

In tests for genetic bottlenecks, we employed five models of evolution; populations were determined to have undergone a recent bottleneck if significant results were obtained for tests using two or more mutation models. Using this criterion, only one population (AR06) showed significant evidence of a genetic bottleneck (S3 Table).

### Analysis of genetic structure

Analyses revealed considerable structuring of genetic variation in *P*. *filiformis*. Pairwise *F*_*ST*_ analysis between all possible pairs of populations were significant for all but three population pairs after sequential Bonferroni correction (range = 0.013–0.63; Table 2). Pairwise *G’*_*ST*_ values showed similar patterns as pairwise *F*_*ST*_, but *G’*_*ST*_ values were greater, ranging from 0.02–0.96 (Table 2). *F*_*ST*_ and *G’*_*ST*_ values were generally lowest within each of the four main geographical areas and increased with increasing geographic distance. *F*_*ST*_ and *G’*_*ST*_ values in *P*. *filiformis* were greatest between the OM-AR populations (AR01-03) and those in all other locations (pairwise *F*_*ST*_ range = 0.481–0.63, pairwise *G’*_*ST*_ range 0.89–0.96; Table 2); indeed, the OM-AR populations were as divergent from the rest of *P*. *filiformis* as *P*. *gracilis* (Table 2). In concordance with the pattern of increasing genetic distance with increasing geographic distance, Mantel tests revealed highly significant isolation by distance in *P*. *filiformis* (P<0.001; S1 Fig)

**Table 2:** Pairwise genetic divergence between populations.

Fst/G’st	AR01	AR02	AR03	AR04	AR05	AR06	MO01	MO02	MO03	MO04	MO05	MO06	MO07	MO08	MO09	MO10	MO11	MO12	MO13	MO14	TX01
AR01		0.05	0.38	0.61	0.61	0.57	0.58	0.57	0.58	0.60	0.56	0.61	0.60	0.56	0.54	0.60	0.58	0.63	0.55	0.55	0.547
AR02	0.08		0.37	0.59	0.59	0.55	0.56	0.55	0.56	0.58	0.54	0.59	0.58	0.54	0.52	0.59	0.56	0.60	0.53	0.53	0.507
AR03	0.59	0.58		0.53	0.53	0.51	0.51	0.50	0.51	0.55	0.51	0.55	0.54	0.50	0.48	0.54	0.52	0.55	0.49	0.51	0.481
AR04	0.94	0.93	0.92		0.18	0.45	0.29	0.27	0.31	0.36	0.38	0.45	0.36	0.30	0.28	0.34	0.31	0.38	0.26	0.33	0.395
AR05	0.95	0.95	0.92	0.32		0.39	0.33	0.33	0.37	0.40	0.32	0.39	0.41	0.35	0.34	0.39	0.36	0.41	0.33	0.37	0.417
AR06	0.91	0.90	0.92	0.81	0.71		0.34	0.34	0.38	0.40	0.29	0.38	0.34	0.32	0.33	0.36	0.34	0.36	0.32	0.34	0.413
MO01	0.95	0.95	0.96	0.55	0.64	0.67		0.03	0.17	0.16	0.23	0.33	0.14	0.10	0.11	0.07	0.04	0.15	0.11	0.13	0.33
MO02	0.95	0.94	0.94	0.51	0.63	0.66	0.06		0.12	0.12	0.22	0.32	0.10	0.07	0.06	0.07	0.05	0.12	0.06	0.10	0.312
MO03	0.93	0.92	0.91	0.57	0.67	0.71	0.33	0.23		0.07	0.22	0.31	0.15	0.09	0.06	0.17	0.15	0.15	0.06	0.12	0.352
MO04	0.93	0.92	0.94	0.62	0.71	0.72	0.31	0.23	0.12		0.22	0.28	0.14	0.09	0.05	0.15	0.12	0.13	0.07	0.12	0.374
MO05	0.91	0.90	0.93	0.70	0.59	0.55	0.46	0.44	0.42	0.40		0.18	0.23	0.18	0.18	0.24	0.22	0.20	0.19	0.19	0.387
MO06	0.94	0.93	0.96	0.78	0.69	0.68	0.62	0.60	0.55	0.49	0.33		0.30	0.24	0.24	0.35	0.31	0.24	0.26	0.23	0.418
MO07	0.94	0.93	0.96	0.64	0.73	0.63	0.26	0.18	0.28	0.25	0.42	0.52		0.04	0.10	0.15	0.11	0.05	0.10	0.05	0.363
MO08	0.91	0.91	0.93	0.56	0.66	0.62	0.21	0.15	0.17	0.16	0.35	0.44	0.07		0.06	0.13	0.08	**0.02**	0.06	**0.01**	0.336
MO09	0.92	0.91	0.93	0.54	0.66	0.68	0.23	0.14	0.12	0.10	0.38	0.47	0.20	0.13		0.13	0.10	0.10	0.01	0.08	0.288
MO10	0.96	0.96	0.96	0.62	0.71	0.67	0.14	0.14	0.33	0.26	0.46	0.63	0.27	0.26	0.25		0.03	0.19	0.12	0.17	0.358
	0.95	0.94	0.95	0.58	0.67	0.65	0.09	0.09	0.29	0.23	0.42	0.56	0.21	0.16	0.20	0.06		0.13	0.09	0.11	0.328
MO12	0.92	0.92	0.95	0.66	0.73	0.67	0.30	0.23	0.27	0.22	0.39	0.42	0.09	0.03	0.21	0.36	0.26		0.09	**0.03**	0.358
MO13	0.93	0.92	0.93	0.50	0.64	0.64	0.23	0.13	0.13	0.13	0.39	0.51	0.19	0.12	0.03	0.23	0.19	0.19		0.09	0.289
MO14	0.90	0.89	0.93	0.61	0.70	0.66	0.26	0.21	0.23	0.23	0.38	0.43	0.10	0.02	0.17	0.33	0.22	0.06	0.19		0.329
TX01	**	**	**	**	**	**	**	**	**	**	**	**	**	**	**	**	**	**	**	**	

** Missing data prevented calculation of *G’*_*ST*_ values for *Physaria gracilis* (TX01).

Pairwise *F*_*ST*_ (upper diagonal) and *G’*_*ST*_ (lower diagonal) values for the 21 populations of *P*. *filiformis* within the study. Bold numbers indicate non-significant pairwise *F*_*ST*_ values (P>0.05).

**Fig 2:**
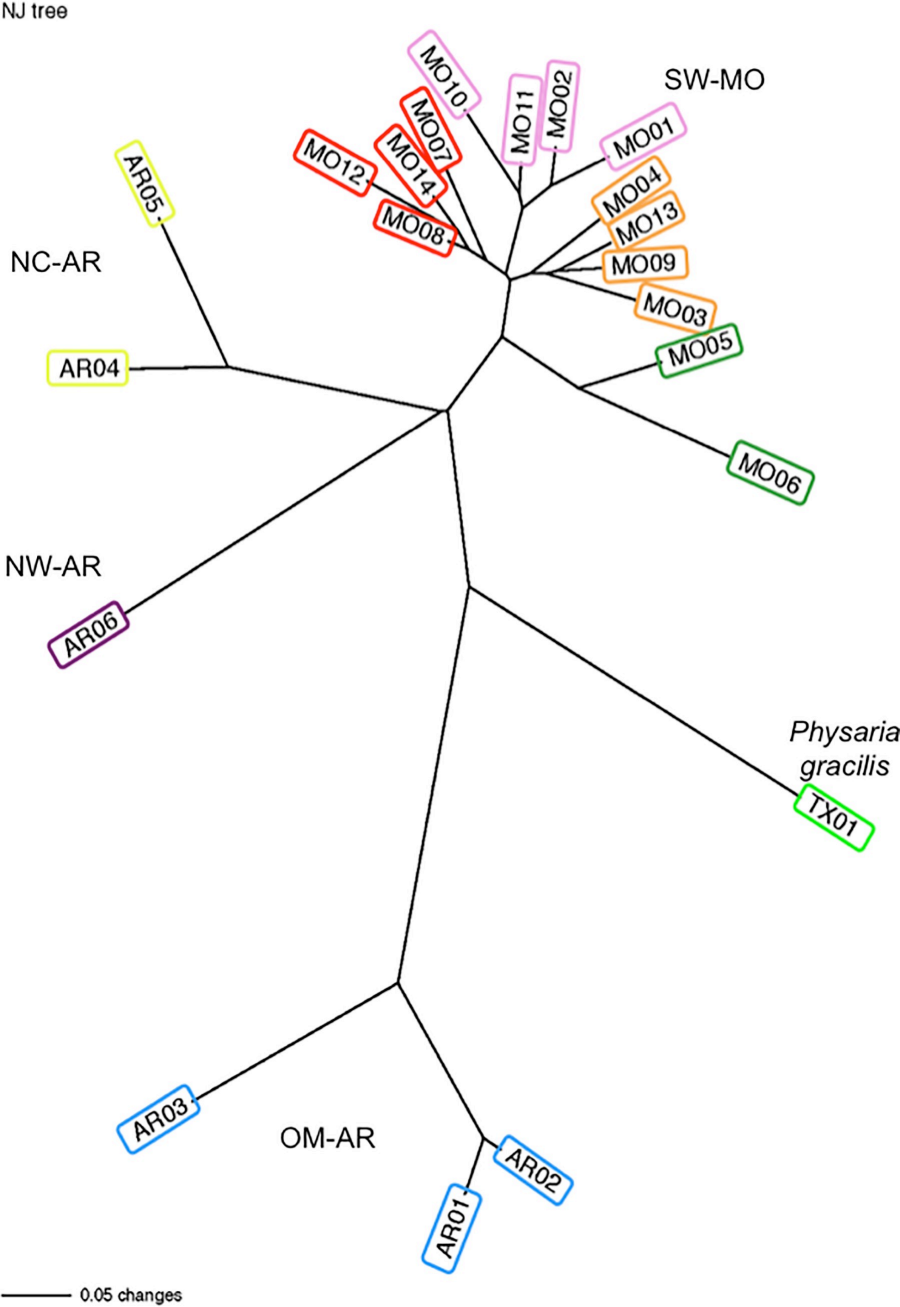
Neighbor joining phylogram based on Nei’s genetic distance. Populations from each of the four main geographical areas of *P*. *filiformis* and *P*. *gracilis* are indicated. Colored boxes around each population label correspond to the main clusters identified using STRUCTURE (Fig 1C).

Correspondingly, the neighbor-joining tree of pairwise genetic distance generally grouped populations according to geography (Fig 2), with populations in the four main geographical areas grouping separately, with each group separated by long branches. All of the populations in SW-MO clustered together, and within this group, populations were divided into four branches reflecting geographic groups (Fig 2). The OM-AR populations were clustered more closely to *P*. *gracilis* than to the other populations of *P*. *filiformis* and were more genetically divergent from the remaining populations of *P*. *filiformis* than was *P*. *gracilis*.

STRUCTURE was used to assess genetic structure without *a priori* assignment of individuals into clusters. Examination of the plot of–ln likelihood values at each *K* showed values plateauing at *K* = 8 (S2 Fig). Examination of results at *K* = 8 revealed clear assignment of individuals to clusters (Fig 1B) by sampling location and geography, congruent with the groups found in the genetic distance tree (Fig 2). The genetic clusters at K = 8 were: (1) the three OM-AR populations (AR01–03), (2) the two NC-AR populations (AR04–05), (3) the NW-AR population (AR06), (4) the southernmost MO populations near Wilson’s Creek National Battlefield (MO01, 02, 10 and 11), (5) individuals in the populations in the northwest part of the species range in MO (MO03, 04, 09, and 13), (6) individuals in two populations to the south and west of Willard, MO (MO05–06), (7) individuals in populations just to the North or West of Rocky Barrens near Willard, MO (MO03, 04, 09 and 13), and (8) all individuals of *Physaria gracilis* (TX1) (Fig 1B; Table 1). Interestingly, at lower K values (K = 1–4), STRUCTURE clustered the OM-AR with *P*. *gracilis* (S3 Fig), consistent with the close relationship found between them in the genetic distance tree (Fig 2).

AMOVA was used to investigate how genetic variation was partitioned within and among populations and to compare four different population-grouping strategies (physiographic region, soil substrate, the four geographic areas, or the STRUCTURE clusters) in capturing among-population genetic variation. Regardless of how populations were grouped, the majority of the variation in *P*. *filiformis* was found within populations (~50%). Less variation was partitioned among groups (~40%) and very little was found among populations within groups (Table 3). When comparing the three different grouping strategies, the greatest percentage of among-group genetic variation was recovered when groups were divided based on physiographic region (41.8%), reflecting the large genetic divergence between the OM-AR populations and all others. The population groupings based on the four main geographical areas explained slightly less among-group variation (39.3%), followed closely by the grouping by soil substrate (38.4%) Table 3); the latter two grouping strategies showed similar amounts of among-population variation because geography and soil are highly correlated variables in *P*. *filiformis* and the grouping schemes differ only slightly (Table 1). Finally, the STRUCTURE grouping scheme explained the lowest amount of among-group variation (29.3%; Table 3).

**Table 3:** Comparative AMOVA analysis of hierarchical population structure.

	Source of Variation	Sum of Squares	Variance Components	Percentage Variation	Fixation Indices
Physiographic region	Among Groups	400.5	3.2	41.8	*F*_*CT*_: 0.42
	Among Populations Within Groups	566.7	1.3	17.0	*F*_*SC*_: 0.29
	Within Populations	1321.1	3.1	41.2	*F*_*ST*_:0.58
Substrate	Among Groups	534.7	2.6	38.4	*F*_*CT*_: 0.38
	Among Populations Within Groups	436.7	1.0	15.3	*F*_*SC*_: 0.25
	Within Populations	1318.3	3.13	46.3	*F*_*ST*_: 0.53
Four main geographic regions	Among Groups	626.6	2.6	39.3	*F*_*CT*_: 0.39
	Among Populations Within Groups	340.7	0.83	12.73	*F*_*SC*_: 0.39
	Within Populations	1321.1	3.14	47.92	*F*_*ST*_: 0.52
Structure	Among Groups	718.3	1.6	29.3	*F*_*CT*_: 0.29
	Among Populations Within Groups	253.1	0.8	13.8	*F*_*SC*_: 0.19
	Within Populations	318.3	3.1	56.9	*F*_*CT*_: 0.43

A comparison of AMOVA results when populations were grouped in four different ways: Physiographic area (Ozark Plateau vs. Ouachita mountains), soil substrate (as defined in Table 1), geographical location (as defined in Table 1), and Structure clusters (K = 8). The population of *Physaria gracilis* was removed in all AMOVA analyses.

## Discussion

### Levels of genetic diversity within populations of *P*. *filiformis*

Overall, levels of genetic diversity in *P*. *filiformis* were high. Levels of genetic diversity in *P*. *filiformis* were comparable or greater to those found in the congener *P*. *gracilis*. It is also worth noting that levels of genetic diversity in *P*. *filiformis* were similar to [76] or greater than [77–79] those found in many other rare, self-incompatible species in the Brassicaceae family, although comparisons of genetic diversity to these other species are somewhat limited because they employed different molecular markers. Furthermore, most populations of *P*. *filiformis* had consistently high numbers of alleles, allelic richness, and observed and expected heterozygosity with few populations showing evidence of reductions in genetic diversity due to factors associated with small population size. Populations had inbreeding coefficients that were close to zero, consistent with the results of a previous pollination study [42] that concluded that *P*. *filiformis* is self-incompatible and has a broad and generalist pollination system that is highly efficient at ensuring cross-pollination. Previous studies of *P*. *filiformis* also generally found high genetic diversity within populations and a lack of evidence for inbreeding [46–49].

In contrast to the majority of populations of *P*. *filiformis*, three populations, none of which have been the subject of a previous genetic analysis, showed evidence of reductions in genetic diversity from either a genetic bottleneck or drift. The lone population in NW-AR (AR06) showed significant evidence of a genetic bottleneck. Interestingly, the AR06 population was discovered recently and had not been managed, but the implementation of management practices such as land clearing and burning has recently resulted in an increase in population size (P. McKenzie, pers. comm), which may explain the observed bottleneck. In addition, the two OM-AR populations showed lower-than-average alleles per locus and allelic richness, which may be the result of random genetic drift eroding genetic diversity in these two populations. This is particularly notable because the OM-AR populations are very genetically divergent from the rest of *P*. *filiformis* and may represent a new, very rare species (see below); these results indicate that these populations may be in need of management to avoid further losses in genetic diversity.

### Structuring of genetic variation

Genetic structure is created by limited migration among populations and genetic drift and different selection regimes acting across populations [80]. Genetic structure in plants is shaped in part by life-history traits such as mating system, population density, pollinator and seed dispersal mechanism, and continuity of geographical distribution [12,13,15,20,24,81]; for example, plant populations that are spaced farther than the distances that pollinators and seed dispersers can travel may have restricted migration, leading to genetic structure. *P*. *filiformis* has a naturally fragmented distribution corresponding to the availability of glade habitats, which occur on rock outcrops that have a patchy distribution across the study area. *Physaria filiformis* is pollinated by a generalist cohort of insects and its seeds are gravity-dispersed [12,13], neither of which is capable of facilitating movement over more than a few kilometers. Concordant with its patchy distribution and low capacity for migration, we observed strong patterns of genetic structure in *P*. *filiformis*. For example, STRUCTURE divided individuals of *P*. *filiformis* into seven major clusters (with the eighth cluster comprised of *P*. *gracilis*), with each cluster encompassing populations occurring within a relatively small geographic area, particularly within SW-MO. The species also exhibited significant isolation by distance, indicating that migration is geographically limited [82]. The strong genetic structure in *P*. *filiformis* suggest that its insect pollinators and gravity seed dispersal are not only incapable of moving pollen or seeds across the distances that separate the four main population groups, but also between geographically proximal areas within SW-MO. Consistent with these results, a previous study on another glade-endemic plant in the region also showed very strong patterns of genetic structure [83]. Additional studies on other plant species are needed to understand whether this is a common characteristic of glade plants in the region.

Interestingly, the geographic distance separating the population groups may have resulted in cryptic speciation. The greatest among-population variation in AMOVA analyses was explained when populations were separated based on physiographic region, with the Ouachita populations separated from the remaining populations that occupy the Ozark Plateau [37]. The genetic distance and pairwise *F*_*ST*_ and *G’*_*ST*_ analyses also revealed that the OM-AR (AR01-AR03) populations were highly genetically divergent from all other populations of *P*. *filiformis* and are roughly as divergent from the rest of *P*. *filiformis* as the morphologically and ecologically distinct congener, *P*. *gracilis*. The STRUCTURE results and genetic distance tree also indicated that the OM-AR populations may be more closely related to *P*. *gracilis* than to *P*. *filiformis*, although additional phylogenetic analysis is needed to confirm this result. This was surprising, as the Ouachita populations are very morphologically similar to *P*. *filiformis*. Given the magnitude of the pairwise *F*_*ST*_ and *G’*_*ST*_ values, it is highly unlikely that any recent gene flow has occurred between the OM-AR populations and the rest of *P*. *filiformis*. The OM-AR populations and the populations occupying the Ozark plateau are separated by the Boston Mountains and the Arkansas Valley, and these physical barriers appear to have prevented gene flow, as was found previously in other organismal groups distributed in both the Ouachitas and Ozark Plateau [84].

Several factors suggest that the populations in the Ouachita Mountains may comprise a previously unrecognized, extremely rare, cryptic species. In addition to being geographically and genetically distinct, the populations in the Ouachita Mountains are ecologically divergent because they occupy sites with shale substrates, whereas the Ozark Plateau populations occur on calcareous substrates. Whether adaptation to substrate differences has contributed to the divergence among these groups is unknown, but merits future study. However, even though this putative new species can be differentiated based on substrate, geography, and genetics, we feel that it is important to be able to differentiate a species based solely on morphological observations; we are therefore currently conducting a morphological study to identify features that could be used to differentiate them from *P*. *filiformis*.

### Conservation recommendations

If the Ouachita populations are recognized as a unique species, then this will have several implications for conservation. If it is a species, then additional research will be needed to determine its conservation status. Currently, it is known from only three populations and additional field surveys in the Ouachita Mountains will be needed to determine whether additional populations exist. Whether the Ouachita populations are recognized as a unique species is also important for *P*. *filiformis*, because it would result in a decrease for *P*. *filiformis* in both the known number of populations and its geographic range size. Thus, determining the taxonomic status of this potential new species is critical for conservation of both it and *P*. *filiformis*. However, regardless of whether the Ouachita populations are recognized as a distinct species, they undoubtedly represent an Evolutionarily Significant Unit (ESU [85]) and it will be important to ensure their conservation. Similarly, the three other main population localities (SW-MO, NW-AR and NC-AR) are highly genetically differentiated and merit recognition as ESUs. Ensuring the protection of populations in each of these regions should be priorities for conservation.

Results of STRUCTURE also showed that genetic diversity in *P*. *filiformis* is structured at an even finer geographic scale than represented by the four main geographic groups, particularly in SW-MO;, to protect as much genetic variation in the species as possible with limited resources, conservation efforts should therefore focus on conserving at least one population within each genetic group of *P*. *filiformis*. At least one population is publicly protected for six of the seven genetic clusters identified by STRUCTURE (Table 1). The genetic cluster that is currently unprotected comprises the two populations from NC-AR (AR04–05) indicated in yellow in Fig 1A (i.e., group B in Table 1). Any future efforts to acquire or protect additional populations of *P*. *filiformis* should prioritize these populations. In addition, integrating results of the present study with future climate projections for *P*. *filiformis* would be useful to ensure that any areas targeted for conservation have both high genetic diversity and will maintain suitable climatic conditions in the future.

For *ex situ* conservation, we considered both genetic structure and private alleles to devise a population prioritization scheme (Table 1); we recommend collecting seed from at least one population per genetic cluster, with priority given to the population within each cluster that has the greatest number of private alleles. Some of the genetic clusters are already represented in the Missouri Botanical Garden’s conservation seed bank, most of which were collected from protected populations. Although we attempted to collect seed from priority populations in 2016 and 2017, these efforts were unsuccessful because no individuals successfully set seed in the populations that we visited. Seed set is highly variable across years, likely varying in response to environmental variation. Thus, future seed banking efforts may need to be timed to coincide with mass flowering events.

Along with public protection of the NC-AR population group and additional seed banking of priority populations, we also recommend additional conservation actions for the species such as monitoring and land management. Through fieldwork to collect genetic samples, we found that *P*. *filiformis* was absent or only represented by a single individual in 11 of the 25 populations that we visited in MO in 2015 and 2016. This does not necessarily mean that the species has been extirpated from these sites; although no formal study of the longevity of *P*. *filiformis* seeds has been conducted, anecdotal reports suggest that the species may emerge after the clearing or burning of sites in which it had never been observed previously, suggesting that the seeds may have considerable longevity in the soil. However, the absence of *P*. *filiformis* from a large number of sites does indicate a major need for land management. The sites where the species was absent were privately owned and were not well managed to promote the persistence of *P*. *filiformis*, with most sites in need of prescribed fire, thinning of woody vegetation, and control of exotic species, especially cool season exotic grasses (i.e., *Bromus inermis*). However, even sites where plants were present invariably maintained smaller populations than those observed 5–10 years ago. It is possible that this reflects the normal year-to-year fluctuations in population size observed previously in *P*. *filiformis* and that environmental conditions will be favorable for *P*. *filiformis* in future years. However, it is also possible that this is indicative of an overall decline in the species in MO. To distinguish whether the species is declining or simply demonstrating normal population fluctuations, we recommend more frequent population monitoring to track population trends [86]. The species would also benefit from a longitudinal ecological study to identify the climate conditions that are favorable for its growth. An increased effort to conduct land management activities to promote the growth of *P*. *filiformis*, such as removing cedars and conducting late summer or early fall prescribed burns [37], would also be beneficial in several publicly protected sites.

## Supporting information

S1 Fig Scatter plot of geographic distance versus genetic distance. (TIF)

S2 Fig Plot of -ln likelihood for each value of *K*. Values begin to plateau at *K* = 8.
(DOCX)

S3 Fig Results of STRUCTURE analyses for *P*. *filiformis and P*. *gracilis* individuals at *K* = 2–8. Populations are separated by black lines. Each vertical line within a population represents an individual, the genetic clusters are represented by a unique color, and the proportion of membership of each individual in genetic clusters is indicated by the colors of each line. The four main geographic regions are indicated at the bottom of. OM-AR, Ouachita Mountains, Arkansas; NC-AR, north-central Arkansas; NW-AR, northwestern Arkansas; SW-MO, southwestern Missouri.
(TIF)

S1 Table Sample collection information. Collection information for each population sampled in the study, including the population identifier used in the study, county, geographic area, locality, latitude and longitude (with the number of coordinates truncated to protect the species), soil type, conservation status, and voucher information.
(DOCX)

S2 Table Information about the microsatellite loci developed in this study for *Physaria filiformis*. Includes primer sequences (forward and reverse), repeat motif, and the size range of alleles as determined in 8 individuals sampled from across the range of the species.
(DOCX)

S3 Table Results of bottleneck analysis. Analyses of *P*. *filiformis* and *P*. *gracilis* were conducted using five different models of evolution: The infinite alleles model (IAM), the stepwise mutation model (SMM), or the two-phase mutation Model (TPM) with 70, 80, and 90% of the loci assumed to be following the SMM. Values are the significance value of the one-tailed Wilcoxon signed rank test for heterozygote excess, with bold values indicating those that are significant (P<0.05).
(DOCX)

S1 File Raw microsatellite data at 16 microsatellite loci for all 480 individuals included in the study. Data in single-row STRUCTURE format. The locus names are shown in the first row with all other rows showing data for each individual. The first column indicates the name of each individual with the names corresponding to the populations listed in Table 1. The second column indicates population and the third column shows the “USEPOPINFO” flag set to 0 indicating that population information should not be used in the analysis. All other columns showing data with the loci in the order listed in row 1, with two columns per locus. Missing data indicated by “-9”.
(TXT)
