# Supplementary figures and images for: Conservation genetics of the threatened plant species Physaria filiformis (Missouri bladderpod) reveals strong genetic structure and a possible cryptic species
Source: PLoS One. 2021 Mar 11;16(3):e0247586. doi: 10.1371/journal.pone.0247586 (PMC7951829; doi:10.1371/journal.pone.0247586)

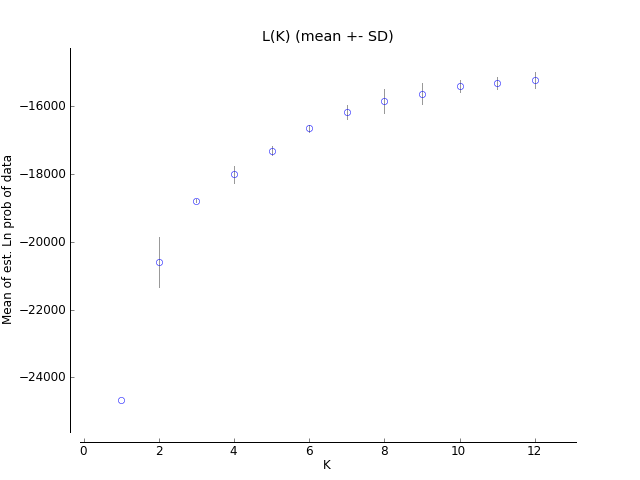


**S1 Figure.** Plot of ln-likelihood for each value of *K*. Values begin to plateau at *K* = 8.

Supplement: S2 Fig — Values begin to plateau at K = 8. (DOCX) [file pone.0247586.s002.docx]

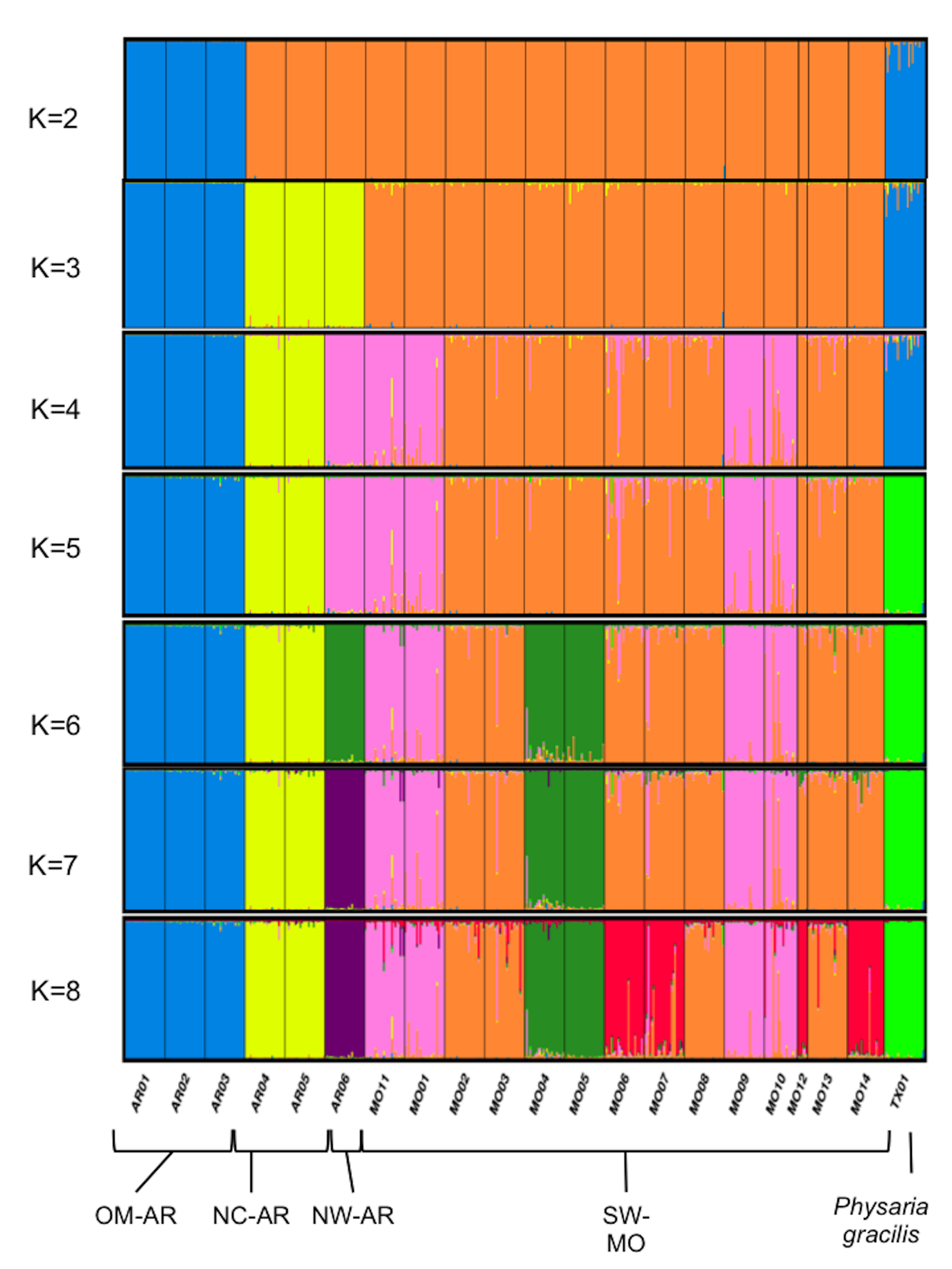

Supplement: S3 Fig — Populations are separated by black lines. Each vertical line within a population represents an individual, the genetic clusters are represented by a unique color, and the proportion of membership of each individual in genetic clusters is indicated by the colors of each line. The four main geographic regions are indicated at the bottom of. OM-AR, Ouachita Mountains, Arkansas; NC-AR, north-central Arkansas; NW-AR, northwestern Arkansas; SW-MO, southwestern Missouri. (TIF) [file pone.0247586.s003.tif]
