# Supplementary material for: Conservation genetics of the threatened plant species Physaria filiformis (Missouri bladderpod) reveals strong genetic structure and a possible cryptic species
Source: PLoS One. 2021 Mar 11;16(3):e0247586. doi: 10.1371/journal.pone.0247586 (PMC7951829; doi:10.1371/journal.pone.0247586)
Supplement: S1 Table — Collection information for each population sampled in the study, including the population identifier used in the study, county, geographic area, locality, latitude and longitude (with the number of coordinates truncated to protect the species), soil type, conservation status, and voucher information. (DOCX) [file pone.0247586.s004.docx]

**S1 Table.** **Sample collection information.** Collection information for each population sampled in the study, including the population identifier used in the study, county, geographic area, locality, latitude and longitude (with the number of coordinates truncated to protect the species), soil type, conservation status, and voucher information..

| Pop ID | County | Geographic area* | Locality | Lat | Long | Soil Type | Publicly protected | Voucher # (Herbarium) | | | |
| --- | --- | --- | --- | --- | --- | --- | --- | --- | --- | --- | --- |
| *Physaria filiformis* | |  |  |  |  |  |  | |  |  |  |
| AR01 | Garland | OM-AR | Cedar Fourche Recreation Area | 34.67 | -93.28 | Shale | yes | Yatskievych 15-003 (MO) | | | |
| AR02 | Garland | OM-AR | North side of Lake Ouachita | 34.66 | -93.27 | Shale | yes | Yatskievych 15-009 (MO) | | | |
| AR03 | Hot Spring | OM-AR | Ross Foundation Property | 34.35 | -93.07 | Shale | no | Yatskievych 15-012 (MO) | | | |
| AR04 | Sharp | NC-AR | North side of Evening Shade | 36.10 | -91.63 | Dolomite | no | Yatskievych 15-021 (MO) | | | |
| AR05 | Izard | NC-AR | Hars Creek Road | 36.18 | -91.68 | Dolomite | no | Yatskievych 15-024 (MO) | | | |
| AR06 | Washington | NW-AR | Blue Springs Recreation Area | 36.16 | -94.01 | Limestone | yes | Witsell 15-0120 (MO) | | | |
| MO01 | Christian | SW-MO | Saunders valley glade | 37.09 | -93.35 | Limestone | no | Yatskievych 15-050 (MO) | | | |
| MO02 | Greene | SW-MO | Williams Glade | 37.11 | -93.34 | Limestone | no | Yatskievych 15-051 (MO) | | | |
| MO03 | Dade | SW-MO | Fairview Glade | 37.43 | -93.68 | Limestone | no | Rimer s.n. (MO) | | | |
| MO04 | Lawrence | SW-MO | Misemer Cemetery Glades | 37.20 | -93.70 | Limestone | No | Rimer s.n. (MO) | | | |
| MO05 | Greene | SW-MO | Highway O Glade | 37.31 | -93.37 | Limestone | No | Rimer s.n. (MO) | | | |
| MO06 | Greene | SW-MO | Seepside Glade–Willard Quarry | 37.30 | -93.41 | Limestone | No | Rimer s.n. (MO) | | | |
| MO07 | Greene | SW-MO | Bois D’Arc Conservation Area | 37.29 | -93.49 | Limestone | Yes | Rimer s.n. (MO) | | | |
| MO08 | Greene | SW-MO | Rocky Barrens Conservation Area | 37.31 | -93.40 | Limestone | Yes | Rimer s.n. (MO) | | | |
| MO09 | Greene | SW-MO | Two Horse Glade | 37.34 | -93.56 | Limestone | No | Rimer s.n. (MO) | | | |
| MO10 | Greene | SW-MO | Wilsons Creek - Wire Road | 37.09 | -93.42 | Limestone | Yes | Edwards 281 | | | |
| MO11 | Greene | SW-MO | Wilsons Creek - Bloody Hill | 37.10 | -93.41 | Limestone | Yes | Edwards 282 | | | |
| MO12 | Greene | SW-MO | Quinn Glade | 37.34 | -86.81 | Limestone | No | Edwards 284 (MO) | | | |
| MO13 | Greene | SW-MO | Nathan Boone State Historic Site | 37.35 | -93.55 | Limestone | Yes | Edwards 286 (MO) | | | |
| MO14 | Greene | SW-MO | Rocky Barrens - Wester Glade | 37.32 | -93.40 | Limestone | Yes | Rimer 18511 (MO) | | | |
| *Physaria gracilis* | |  |  |  |  |  |  | |  |  |  |
| TX01 | Williamson | TX | San Gabriel River | 30.66 | -97.42 | Mudstone |  | Yatskievych 16-110 (TEX) | | | |

*OM-AR, Ouachita Mountains, Arkansas; NC-AR, north-central Arkansas; NW-AR, northwestern Arkansas; SW-MO, southwestern Missouri.
