# Supplementary material for: Conservation genetics of the threatened plant species Physaria filiformis (Missouri bladderpod) reveals strong genetic structure and a possible cryptic species
Source: PLoS One. 2021 Mar 11;16(3):e0247586. doi: 10.1371/journal.pone.0247586 (PMC7951829; doi:10.1371/journal.pone.0247586)
Supplement: S2 Table — Includes primer sequences (forward and reverse), repeat motif, and the size range of alleles as determined in 8 individuals sampled from across the range of the species. (DOCX) [file pone.0247586.s005.docx]

**S2 Table** Information about the microsatellite loci developed in this study for *Physaria filiformis*. Includes primer sequences (forward and reverse), repeat motif, and the size range of alleles as determined in 8 individuals sampled from across the range of the species.

| Locus name | Forward primer sequence | Reverse primer Sequence | Repeat Motif | Size Range |
| --- | --- | --- | --- | --- |
| PF8 | *ACCAATTGCAGATGAAGGCG | AGTTCTTTGTTTCGAGGGTCC | AG(10) | 183-194 |
| PF26 | *TTGTAGGTGTGGCTTCGTTG | TCATCTCCACGCTCACTGAG | AG(9) | 182-186 |
| PF33 | *CCAGATTCCGACACGACTTG | AACCCACATCCCACGCATC | AG(9) | 113-131 |
| PF37 | *GCTTCGTATTCCTGATGCCG | CGTTTACCAAATCACTGCGC | AG(9) | 147-160 |
| PF49 | *ATAGTAAGCGGACCGAACCC | AACCCTCGTAACACAGAGCG | AG(20) | 119-152 |
| PF57 | *TGCTAACACAGAGACCGGG | ACTCCCAAGTCCCATCCAC | AC(8) | 115-128 |
| PF58 | *GAAGCAGACGAAGGGACAAC | GCAATTGATTAGCGAAACCGG | AG(12) | 149-163 |
| PF62 | *ACCTTCTTCGTCATCTCCGG | CATCTCCGCACCTTTACACC | AG(8) | 136-142 |
| PF79 | *TCGTCGCTAGGAAGATCGG | GACTTCCACGGTCGGGTATC | AG(11) | 173-184 |
| PF82 | *TCCTCCACCGTCAAGTCATC | CACACACCATCTTCTTTGCAC | AG(14) | 141-156 |
| PF84 | *TCAAACGTAGCCTGCAAAGC | TGAGCAAGATGATGTGATAGCC | AT(8) | 174-184 |
| PF88 | *AGGTGATGTTCTCGTTTGCC | CATACCAAAGACCTTCCAAAGC | AT(8) | 139-143 |
| PF93 | *GAAGATGTTTAGCCGCTGGG | CGTTGACCGTGAAGATGTGAG | AG(10) | 113-123 |
| PF103 | *CGCCACTCTTGTTCTCACC | CAGGTATGTTCCGAAGCGTTAG | AAG(9) | 111-136 |
| PF121 | *CAAGTCCCTGATGAAGAACCC | AGTGTCGGTCTTCTCTTTCG | AAAT(6) | 135-145 |
| PF122 | *ACGCAATGGAGAAGCAAAGG | ATAAGAGTGTGGTCCAGGCC | AAG(8) | 120-129 |

*M13 tag (CACGACGTTGTAAAACGAC) added to 3′ end of primer for amplification using a universal dye-labeling approach.
