# Supplementary material for: Conservation genetics of the threatened plant species Physaria filiformis (Missouri bladderpod) reveals strong genetic structure and a possible cryptic species
Source: PLoS One. 2021 Mar 11;16(3):e0247586. doi: 10.1371/journal.pone.0247586 (PMC7951829; doi:10.1371/journal.pone.0247586)
Supplement: S3 Table — Analyses of P. filiformis and P. gracilis were conducted using five different models of evolution: The infinite alleles model (IAM), the stepwise mutation model (SMM), or the two-phase mutation Model (TPM) with 70, 80, and 90% of the loci assumed to be following the SMM. Values are the significance value of the one-tailed Wilcoxon signed rank test for heterozygote excess, with bold values indicating those that are significant (P<0.05). (DOCX) [file pone.0247586.s006.docx]

**S4 Table Results of Bottleneck analysis.** Analyses of *P. filiformis* and *P. gracilis* were conducted using five different models of evolution: the infinite alleles model (IAM), the stepwise mutation model (SMM), or the two-phase mutation Model (TPM) with 70, 80, and 90% of the loci assumed to be following the SMM. Values are the significance value of the one-tailed Wilcoxon signed rank test for heterozygote excess, with bold values indicating those that are significant (P<0.05).

| Population | IAM | SMM | TPM | | |
| --- | --- | --- | --- | --- | --- |
|  |  |  | 70% | 80% | 90% |
| AR01 | 0.087 | 0.517 | 0.350 | 0.350 | 0.382 |
| AR02 | 0.271 | 0.879 | 0.596 | 0.709 | 0.768 |
| AR03 | 0.073 | 0.729 | 0.294 | 0.368 | 0.500 |
| AR04 | **0.026** | 0.485 | 0.212 | 0.259 | 0.367 |
| AR05 | **0.026** | 0.961 | 0.339 | 0.425 | 0.788 |
| **AR06*** | **0.0004** | 0.342 | **0.029** | 0.055 | 0.095 |
| MO11 | 0.148 | 0.966 | 0.687 | 0.749 | 0.914 |
| MO01 | 0.068 | 0.874 | 0.262 | 0.577 | 0.719 |
| MO02 | **0.025** | 0.665 | 0.179 | 0.232 | 0.380 |
| MO03 | 0.404 | 0.992 | 0.923 | 0.948 | 0.979 |
| MO04 | 0.207 | 0.773 | 0.446 | 0.554 | 0.658 |
| MO05 | **0.029** | 0.966 | 0.271 | 0.473 | 0.773 |
| MO06 | 0.134 | 0.903 | 0.572 | 0.665 | 0.805 |
| MO07 | 0.207 | 0.998 | 0.793 | 0.905 | 0.960 |
| MO08 | 0.121 | 0.997 | 0.787 | 0.892 | 0.955 |
| MO09 | 0.084 | 0.896 | 0.533 | 0.619 | 0.700 |
| MO10 | **0.0002** | 0.604 | 0.065 | 0.117 | 0.190 |
| MO12 | 0.249 | 0.847 | 0.632 | 0.706 | 0.773 |
| MO13 | **0.016** | 0.892 | 0.188 | 0.368 | 0.607 |
| MO14 | 0.077 | 0.932 | 0.500 | 0.620 | 0.729 |
| TX01 | **0.003** | 0.658 | 0.055 | 0.084 | 0.318 |

*Indicates populations considered to demonstrate a significant bottleneck because of significant tests using two or more mutation models
